# Medullary thyroid carcinoma with double negative calcitonin and CEA: a case report and update of literature review

**DOI:** 10.1186/s12902-019-0435-7

**Published:** 2019-10-16

**Authors:** Claudio Gambardella, Chiara Offi, Guglielmo Clarizia, Roberto Maria Romano, Immacolata Cozzolino, Marco Montella, Rosa Maria Di Crescenzo, Massimo Mascolo, Angelo Cangiano, Sergio Di Martino, Giancarlo Candela, Giovanni Docimo

**Affiliations:** 1 Division of Thyroid Surgery - Department of Medical and Advanced Surgical Sciences, University of Campania “Luigi Vanvitelli”, School of Medicine, Via Sergio Pansini, 5, 80131 Naples, Italy; 2 Department of Cardiothoracic Sciences, University of Campania “Luigi Vanvitelli”, School of Medicine, Via Sergio Pansini 5, 80131 Naples, Italy; 3 Pathology Unit, Department of Mental and Physical Health and Preventive, Medicine University of Campania “Luigi Vanvitelli”, School of Medicine, Piazza Miraglia 2, 80138 Naples, Italy; 4 Department of Advanced Biomedical Sciences, Pathology Section, University of Naples Federico II, Via Sergio Pansini 5, 80131 Naples, Italy

**Keywords:** Medullary thyroid carcinoma, Thyroid cancer, Calcitonin, Carcinoembryogenic antigen

## Abstract

**Background:**

Medullary thyroid carcinoma is a malignant uncommon and aggressive tumour of the parafollicular C cells. In about 75% of cases it is sporadic while, in case of RET mutation, it is associated to multiple endocrine neoplasia type 2 (25% of cases). The biochemical features of medullary thyroid carcinoma include the production of calcitonin and carcinoembryogenic antigen. The above-mentioned features are useful in the diagnostic process as well as in the follow up and in the prognostication of the disease. Even if calcitonin elevation is strongly associated to MTC, it can also be found increased in many pathological different conditions as pregnancy, lactation, C-cells hyperplasia, autoimmune thyroiditis, end stage renal disease, lung and prostate cancer and several neuroendocrine tumours. Major medullary thyroid tumours are usually connected to high doses of circulating calcitonin, in fact non-secretory variants have hardly been described.

**Case presentation:**

We herein report the case of a 59 years old male, who had undergone total thyroidectomy for multinodular goiter with negative preoperative calcitonin, showing medullary thyroid carcinoma at definitive pathology. To the best of our knowledge, this is the first case documenting a non-secretory medullary thyroid carcinoma, with double negative markers at the time of diagnosis and at the relapse.

**Conclusion:**

A Literature review underlining pathological hypothesis, differential diagnosis and alternative and innovative biomarkers to identify non-secretory medullary thyroid carcinoma was carried out.

## Background

Medullary thyroid carcinoma (MTC) is a malignant thyroid tumour originating from the parafollicular C cells. MTC is an uncommon disease, accounting around 1 to 10% of all thyroid cancers. It is characterised by a mean survival of 8.6 years, and a 10-years survival rates ranging from 69 to 89% [1]. MTC is the second most aggressive thyroidal cancer, after anaplastic carcinoma, accountable of the 14% of all thyroid cancers [1]. It may occur sporadically (75% of cases), otherwise in hereditary form (25% of cases), in case of RET proto-oncogene germline mutation, associated with multiple endocrine neoplasia type 2 (MEN 2) [2]. Sporadic MTC has a low growth rate, it is well differentiated, it generally shows up with local aggressiveness and it is frequently diagnosed at the stage of cervical nodal involvement [1]. Hereditary forms of MTC usually show locoregional invasion and lymph node metastases at the time of diagnosis [3].

Round cells producing amyloid substance, separated by fibrous septa and areas of microcalcification are the histological typical patterns of MTC. As biochemical characteristics, MTC are known for the secretion of calcitonin and carcinoembryogenic antigen (CEA) whose determination is important in the diagnostic phase as well as the follow up and prognosis, other than being related to the specific tumour patterns.

Calcitonin is a 32-amino acid monomeric peptide secreted by C-cells. In MTC, calcitonin is high at basal and after pentagastrin stimulation test and it is considered a highly sensitive and specific indicator of the disease. Early diagnosis of MTC is crucial because surgery is the only curative therapy. Therefore, in case of thyroid nodular disease, it is mandatory to include calcitonin evaluation in the routine work-up, in order to achieve an early MTC diagnosis [4]. Tumoral biomarkers evaluation can be controversial. MTC and NETs in general can in fact produce different markers such as procalcitonin, the precursor of calcitonin, neuron specific enolase (NSE) and chromogranin A (CgA). The same ones can be secreted in several neoplastic or benign conditions or they can be not produced at all, as it happens for millimetric MTC [5, 6]. Voluminous MTC are very rarely found non-secreting, in particular there seems to be a correlation between the calcitonin blood rates and the tumour’s dimensions, though very few calcitonin negative MTC have been described [7].

We herein report the case of a 59 years old male, who had undergone total thyroidectomy for multinodular goiter with negative preoperative calcitonin, showing a MTC at definitive pathology. Moreover, a comprehensive Literature review underlining pathological hypothesis, differential diagnosis, alternative and innovative biomarkers to early identify non-secretory MTC was carried out.

## Case presentation

In June 2014, a 59 years old male with multinodular thyroid goiter was observed in our centre. Due to the presence of thyroidal disease, a hormonal evaluation was performed, showing a slight elevation of FT3 (5.19 pg/mL, normal value 3.5–4.5 pg/mL) and of Thyroglobulin (TG) (> 300 ng/mL, normal value < 50 ng/mL), with FT4, TSH, Ab anti TPO, Ab anti TG and Calcitonin (5.21 pg/ml, normal value < 18.2 pg/ml) resulted in the normal range. Neck ultrasound (US) showed the presence of a large hypoechoic inhomogeneous nodule of 47 mm in the left lobe of thyroid gland, with a retrosternal extension. In the right lobe it was possible to identify only two small nodules, the largest being of 11 mm, with mixed echogenicity. A fine needle aspiration cytology (FNAC) of the left nodule and of the biggest one on the right lobe was performed, both concluding for Thyr 2, negative for malignant cells, according to SIAPEC-IAP classification [8].

**Fig. 1:**
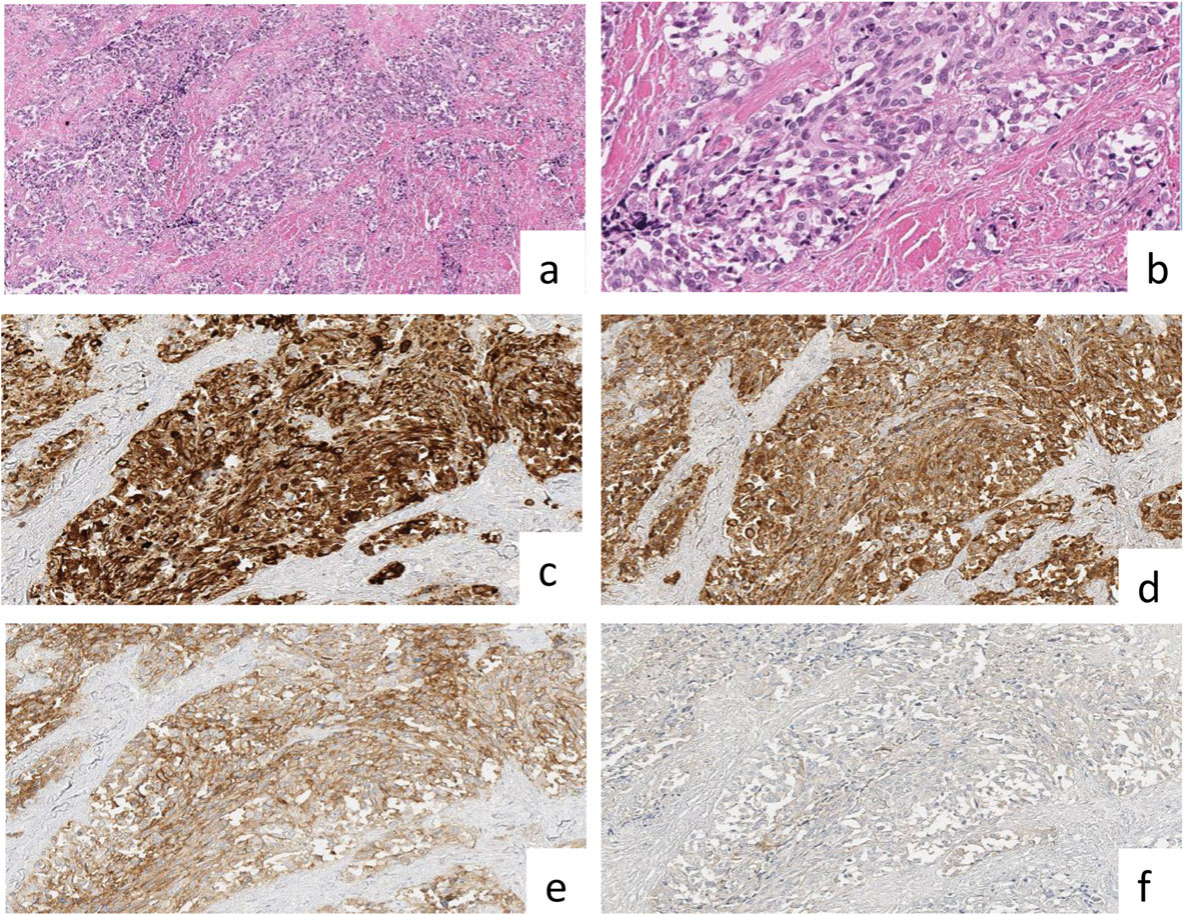
Thyroid definitive pathology with microscopic and immunochemistry evaluation **a**) Low magnification showed nests of neoplastic cells separated by thick septa of fibrous tissue (haematoxylin and eosin, original magnification, x4); **b**) The neoplastic cells have a plasmacytoid appearance (haematoxylin and eosin, original magnification, x20); **c**-**f**) Phenotypic features with positivity for cytokeratin 19 (c: immunoperoxidase stain for anti-cytokeratin 19, original magnification, x 20), calcitonin (**d**: immunoperoxidase stain for anti-calcitonin, original magnification, x 20), weak expression of CD56 (**e**: immunoperoxidase stain for anti-CD56, original magnification, x 20) and negativity for thyroglobulin (**f**: immunoperoxidase stain for anti-thyroglobulin, original magnification, x 20)

After 12 months, due to the worsening of the compressive symptomatology, the patient underwent a total thyroidectomy for a presumed retrosternal goiter at the General and Oncological Surgery Division of University of Campania “Luigi Vanvitelli” of Naples. After 2 days, according to the patient good clinical conditions, the patient was discharged. Despite the good performance status and the presence of no symptoms, definitive pathology showed the presence of a medullary carcinoma of 1.0 × 0.8 cm in the right lobe, while in the left lobe, a follicular adenoma of 6.0 × 4.0 cm, was detected. The right lobe of the thyroid revealed a poorly circumscribed, whitish nodule, measuring 1 cm in its largest diameter. The tumour showed a complex arrangement, which included solid, cord-like and insular areas set in a fibrous struma with relatively few amyloid deposits (Fig. 1a). The neoplastic cells were medium-sized plasmacytoid, round and spindle shaped cells, with abundant eosinophilic cytoplasm and nuclei with clumped chromatin and small nucleoli (Fig. 1b). Scattered mitoses were present. The neoplastic cells expressed cytokeratin 19 (Fig. 1c) and calcitonin (Fig. 1d); a weak reactivity for CD56 (Fig. 1e) and negativity for thyroglobulin (Fig. 1f) were also detected. A diagnosis of medullary thyroid carcinoma was made. Therefore, the patient was introduced to the standard follow up for medullary carcinoma with several and strict evaluations of calcitonin and CEA levels, and neck ultrasound without showing any sign of recurrence. The patient underwent genomic investigations without showing RET proto-oncogene mutation. Moreover,, the patient underwent a total body ^99^Tc scintigraphy in February 2016, in the attempt of precociously identifying a possible recurrence, without showing any areas of abnormal accumulation. In September 2016, during a routine follow-up performed by neck ultrasound, a nodule of 0.8 cm, suspicious for lymph node recurrence, was identified in the right thyroidal space without any elevation of calcitonin (< 2.00 pg/ml) and CEA levels (1.49 ng/ml, normal value < 10). The patient underwent a FNAC of the suspicious lesion, that showed at the morphological examination, on a haematic background, a dispersed neoplastic population, rarely arranged in loosely cohesive groups. The tumour cells were middle-sized cells, with an extremely variable aspect: spindle-shaped elements, epithelioid cells with round nuclei and scanty cytoplasms, sometimes with eccentric nuclei and a “plasmacytoid” appearance, or some voluminous cells with ample granular cytoplasms and pleomorphic and irregular nuclei. The chromatin was finely dispersed with inconspicuous nucleoli. The same morphology was evident on cell-block sections, but unfortunately, the immunocytochemistry investigations were negative for Calcitonin, thyroglobulin and PTH. Nevertheless, the morphological pattern was suggestive for a lymph-nodal recurrence of MTC.

**Fig. 2:**
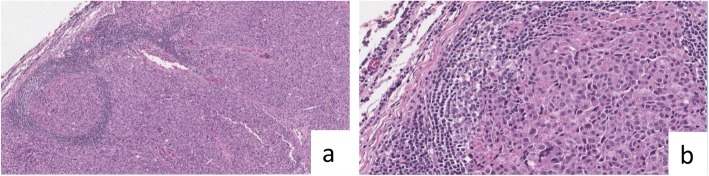
Recurrent laterocervical lymph-node definitive pathology. **a** haematoxylin and eosin, original magnification, × 4. **b** haematoxylin and eosin, original magnification, × 20

Due to the uncertain diagnosis, the patient underwent a total body 18-FDG positron emitting tomography (PET), without showing any metabolic activity. Despite the unconfirmed diagnosis, in February 2017, the patient underwent a central compartment neck dissection. Gross findings of the surgical specimen displayed in a single lymph node of 0.6 cm, the presence of metastasis of medullary thyroid carcinoma (Fig. 2). Similarly, the immunochemistry showed positivity for calcitonin and TTF-1, while thyroglobulin resulted negative.

The patient, did not experience any further biochemical or imaging sign of suspicious recurrence at a 20 months follow-up.

## Discussion and conclusions

We have described the rare case of a 59 years old male with MTC associated with undetectable calcitonin serum level. Only few similar cases have been previously described in Literature (Table 1). To the best of our knowledge, this is the first case documenting a non-secretory MTC, with double negative markers at the time of diagnosis and at the relapse.

**Table 1:** Demographic, clinic-pathological features, treatment, histological diagnosis, follow-up, IHC

*Author*	*Gender*	*Age*	*Size, mm*	*Preoperative calcitonin serum level*	*Calcitonin-HIC*	*Calcitonin-IHC*	*CGRP-IHC*	*CgA-IHC*	*Syn-IHC*	*TG-IHC*	*CEA-IHC*	*RET mutation*	*Follow-up*	*Relapse*
*Sobol*	F	82	20	Normal range	Negative	–	–	+	NA	NA	+	NA	6 month	Lymph nodes, liver and bone
*Schmid 1*	M	28	NA	NA	Weak	NA	+	+	NA	–	–	NA	NA	NA
*Schmid 2*	M	46	NA	NA	Weak	NA	+	+	NA	–	–	NA	19 month	Lymph nodes
*Schmid 3*	F	45	NA	NA	Weak	NA	–	+	NA	–	–	NA	NA	NA
*Schmid 4*	M	37	NA	NA	Negative	NA	–	+	NA	NA	–	NA	NA	NA
*Redding*	F	31	45	28	Diffuse	+	NA	NA	+	NA	+	–	43 month	Negative
*Bockhorn*	F	50	20	0,8	Weak	NA	NA	+	NA	–	+	+	NA	NA
*Sand*	F	73	NA	5,3	Weak	NA	NA	NA	NA	NA	NA	NA	Deceased 6 weeks	NA
*Dora*	M	43	20	4	Diffuse	+		+	+	–	NA	–	NA	NA
*Wang*	M	68	70	38	Weak	+	NA	+	NA	–	+	NA	12 months	Negative
*Giovanella*	F	43	48	4,7	Diffuse	+	NA	NA	NA	NA	NA	NA	24 months	NA
*Alapat*	F	16	30	4	Diffuse	+	NA	+	NA	–	+	NA	20 months	Negative
*Chernyavsky*	F	40	20	2,1	Negative	–	NA	+	+	+	NA	+	12 months	Negative
*Nakazawa*	M	76	60	22	Weak	NA	NA	+	+	–	NA	NA	18 months	Negative
*Frank-Raue 1*	F	61	10	2,9	Weak	+	NA	+	+	–	+	–	72 months	Negative
*Frank-Raue 2*	M	70	80	< 2	Weak	+	NA	+	+	–	+	–	25 months	Pulmonary
*Frank-Raue 3*	F	50	20	0,8	Weak	+	NA	+	+	–	+	+	150 months	Lymph modes
*Frank-Raue 4*	M	47	30	2,6	Focal	+	NA	+	+	–	+	–	18 months	Local tumor infiltration
*Frank-Raue 5*	F	53	45	NA	Diffuse	+	NA	+	+	–	+	+	21 months	Lymph node, bone, brain
*Frank-Raue 6*	M	45	18	11	Weak	+	NA	+	+	–	+	+	21 months	Pulmonary
*Frank-Raue 7*	F	45	55	1,5	Focal	+	NA	+	+	–	+	+	36 months	Pulmonary failure
*Ismi*	NA	57	NA	5,6	Negative	–	NA	+	+	–	NA	NA	NA	NA
*Brutsaert*	F	49	26	< 2	Diffuse	NA	NA	NA	NA	NA	NA	+	NA	NA
*Kim*	M	34	10	3,7	Negative	–	NA	+	+	+	–	–	12 months	Negative
*Kasajima*	F	48	30	29	Negative	NA	+	+	+	NA	NA	–	NA	NA
*Samà 1*	M	60	38	7,8	Focal	+	NA	+	NA	NA	+	–	120 months	Negative
*Samà 2*	F	66	NA	5	NA	NA	NA	NA	NA	NA	NA	NA	120 months	Negative
*Samà 3*	M	53	12	< 10	Negative	–	NA	–	NA	NA	–	+	36 months	Negative
*Samà 4*	M	62	45	13	Focal	+	NA	+	NA	NA	–	–	36 months	Negative
*Parmer*	F	74	20	Normal range	Negative	–	NA	+	+	–	+	NA	NA	NA
*Zhou*	11 M 8 F	≥30 ys 3 cases < 30 ys 16 cases	≤10mmm 14 cases > 10 mm 5 cases	NA	Positive: 8 cases Negative: 11 cases	+ 8 cases - 11 cases	NA	+ 18 cases - 1 case	+ 19 cases	+ 5 cases - 14 cases	+ 4 cases - 15 cases	NA in 15 cases - In 4 cases	NA	NA
*Liu*	F	62	60	2	Weak	+	NA	NA	NA	NA	NA	NA	NA	NA
*Gambardella*	M	59	10	5,21	Weak	+	NA	+	NA	–	NA	–	20 months	Laterocervical Lymh-node

*MTC* medullary thyroid carcinoma, *PDMTC* poorly differentiated MTC, *Und* undetectable, *WDMTC* well differentiated MTC, −, Not Available, *TT* total thyroidectomy, *ET* hemithyroidectomy, *LYA* lymphadenectomy

Basal level of calcitonin above 100 pg/ml request further examinations, as it can help fetching an early diagnosis of MTC, whose importance is clear, as surgery is the only curative treatment for MTC (Table 1). Since elevated calcitonin serum rates can occur in many pathological conditions other than MTC, the differential diagnosis is provided by the pentagastrin stimulating test, showing an increase of calcitonin above 1000 pg/ml in MTC, as well as an associated elevation of CEA and CgA serum levels [9–12]. Calcitonin in FNAC washout fluids, evaluation of serum procalcitonin and calcium stimulation of calcitonin have also been proposed as specific diagnostic tests for MTC [13–15].

FNAC plays a primary role in the management and evaluation of any thyroid nodule, even if it shows a lower diagnostic accuracy for MTC than for other differentiated thyroid carcinomas [16, 17]. The evidence of plasmacytoid and/or spindle shaped cells, salt and pepper like chromatin, dispersed cell pattern, binucleation/ multinucleation, scattered large cells is the pathognominc diagnostic criteria for MTC; its cytology pattern is in fact characterized by the findings of high cellularity with single cells or small clusters, absent colloid, and a variable amount of amyloid substance (positive at Congo-red staining), homogeneous, in rods or spheres (Table 1). Nevertheless, these abovementioned features are rarely co-expressed in the same case, and MTC diagnosis could be suspected if three to four items appeared in the smear [18]. In the reported case, a dispersed neoplastic population, rarely arranged in loosely cohesive groups was identified on a haematic background. The tumour cells were middle-sized cells, with an extremely variable aspect: spindle-shaped elements, epithelioid cells with round nuclei and scanty cytoplasm, sometimes with eccentric nuclei and a “plasmacytoid” appearance, or some voluminous cells with ample granular pleomorphic cytoplasm with inconspicuous nucleoli. These characteristics were different from the appearance of conventional MTC.

To date, only 50 cases of certified “atypical” MTC reported in 20 studies have been described in English Literature, and the current report is the 51th described case (Table 1). Sobol et al. described an 82 years old woman affected by a MTC without calcitonin serum level elevation, but characterized by high CgA serum level, hypothesizing the possibility, in this “chromograninoma”, of an altered co-regulation for genes of CgA and of hormone production [7]. Frank-Raue et al. reported 7 cases of non-secretory MTC, with a prevalence of the 0.83% in his large sporadic MTC population. Moreover, also the latter Authors identified a strong positivity for CgA, suggesting the role of CgA evaluation in addition to CEA in the diagnosis of calcitonin negative MTC [19, 20].

The largest clinical series of non-secretory MTC was reported by Zhou et al., which identified 19 cases of calcitonin negative MTC among their 158 MTC treated patients with a surprisingly high prevalence of 12,02% [21]. Zhou observed larger masses in “typical” MTC group which were also associated with higher rate of lymph nodes metastases, thus identifying tumour size as an independent survival indicator. Moreover, the study suggested a better oncological outcome for non-secretory MTC [21]. Conversely, a bimodal behaviour was reported by Frank-Raue et al., which identified long-term survival patients (12,5 years) or rapid progression diseases (1,75 years), the latter one characterized by over expression of Ki67 and RET gene mutation [20].

Despite the low or undetectable calcitonin serum level of all patients with non-secretory MTC, at IHC in almost the half of the cases reported in Literature, the tumours presented diffuse or focal positivity for calcitonin and CEA, while a CgA positivity in 41 of the 43 tested patients was reported. High CgA serum levels are characteristic of NETs, which share the same neural crest ectoderm derivation similar to parafollicular C-cells with MTC. The correct diagnosis to discriminate between MTC and NETs, is often a challenge due to the presence of spindle-shaped or round cells in trabecular arrangements with the presence of amyloid substance, moreover at IHC staining, both result positive for CgA, NSE and CEA. Therefore, the evaluation of calcitonin serum level and calcitonin at IHC staining are mandatory and diriment [20–23].

Another hard differential diagnosis is with the hyalinaising trabecular tumour, a rare thyroid neoplasm, which shares a similar histological pattern with MTC and a positive immunostaining for CgA, somatostatin and NSE. Conversely, it presents as characteristic and distinctive feature the thyroglobulin hyper-expression [24].

It is of paramount importance to understand the pathophysiology of the MTC to diagnose a calcitonin negative MTC at an early stage and arrive to a differential diagnosis. Several Authors advocated different hypotheses to explain this uncommon non-secretory behaviour. The hook effect, considered as a calcitonin assay interference, is the hypothesis proposed by different Authors [25, 26]. Hook effect or prozone effect occurs when, in a sample, a high level of analytes is present, resulting in a false negative value [27]. Redding et al. have supposed that the “conventional” antibodies would not recognized aberrant calcitonin or precursor molecules produced by abnormal secretory mechanisms and released by tumour cells [28–30]. Another theory appears to be the one proposed by Dora et al., either regarding the capacity of parafollicular tumour cells of synthesizing but not secreting calcitonin; or the total loss of the capacity to codify for it due to a dedifferentiation process [25]. It is also been suggested that some forms of neoplastic C-cells could result calcitonin non-secretive when actually secreting modified proportion of N-pro calcitonin, mature calcitonin and C-pro-calcitonin consequent to an alternative splicing of calcitonin gene related peptide (CGRP), when modern tests only measure monomeric calcitonin [20, 31]. Also, the non-secretory MTC DNA has been studied, calcitonin gene resulted to be mutated, being the reason of an absent or low calcitonin secretion [2]. Nakazawa et al. reached the same conclusion, hypothesizing that low calcitonin serum levels result from epigenetic or/and genetic CT/CGRP gene mutation [32]. Otherwise, since morphologically, MTC cells and thymus carcinoid tumour have the same histological features, Schmid et al. proposed a thymic origin [32–34].

The most debated aspects in non-secretory MTC are diagnosis and follow-up. It is necessary to perform serial imaging tests, for example neck US and computed tomography (CT), liver and chest magnetic resonance (MRI), calcitonin, CEA and CgA serum level evaluations to identify a possible recurrence, even if the imaging methods do not allow the identification of small tumours [25–31]. Otherwise, Frank-Raue et al. have succeeded in demonstrating how selective venous catheterization can identify the 89% of occult persistent MTC [20]. Fluorine 18-fluorodeoxyglucose (18F-FDG) PET/TC has demonstrated its superiority over conventional imaging in identifying relapse or disease persistence, even if many authors do not consider it as a first choice method because of the high costs, the variable sensitivity ranging from 50 to 85% and its inability to detect smaller masses [25–36].

The calcitonin precursor (Procalcitonin) and CGRP have been studied as alternative biomarkers of non-secretory MTC. Procalcitonin in particular, resulted to be comparable to calcitonin in terms of sensitivity and specificity in identifying the primary tumour, the local extension and the eventual presence of metastases [34].

Brutsaert at al reported an elevation of preoperative serum Procalcitonin level in non-secretory MTC patients, with normal serum level after surgery. Procalcitonin is a stable protein with a half-life of 24 h in vivo [31]. With these promising features, Procalcitonin has the characteristics to replace calcitonin in the management of non-secretory MTC. The CALC-A gene that encodes for calcitonin can undergo alternative RNA splicing, producing CGRP. It can be secreted by nervous cells MTC cells and non neoplastic C-cell other than being associated to C-Cell hyperplasia when overexpressed and in co-expression of TTF-1 and PAX-8. An overexpression of CGRP was reported among the metastatic cases more than the primary localizations [31]. Though not fully studied yet, CGRP could be used to differentiate thyroidal NET from non-secretory MTC, as not expressed in follicular cells [35, 37–40].

## Data Availability

The datasets used and/or analysed during the current study are available from the corresponding author on reasonable request. The datasets used and/or analysed during the current study are available from the Division of General Surgery, Second University of Naples, Via Pansini 5 80131 Naples, on reasonable request.

## Abbreviations

18-FDG/PET: 18-FDG positron emitting tomography
CEA: Carcinoembryogenic antigen
CgA: Chromogranin A
CT: Computed tomography
FNAC: Fine needle aspiration cytology
MEN 2: Multiple endocrine neoplasia type 2
MRI): Magnetic resonance
MTC: Medullary thyroid carcinoma
NETs: Neuroendocrine tumours
NSE: Neuron specific enolase
TG: Thyroglobulin
US: Ultrasound
